# Comparison of pathologic outcomes of robotic and open resections for rectal cancer: A systematic review and meta-analysis

**DOI:** 10.1371/journal.pone.0245154

**Published:** 2021-01-13

**Authors:** Yinyin Guo, Yichen Guo, Yanxin Luo, Xia Song, Hui Zhao, Laiyuan Li

**Affiliations:** 1 Lanzhou University Second Hospital, Lanzhou, China; 2 Department of Emergency, The First Hospital of Lanzhou University, Lanzhou, China; 3 Department of Colorectal Surgery, The Sixth Affiliated Hospital of Sun Yat-sen University, Guangzhou, China; 4 Department of Anorectal Surgery, Gansu Provincial Hospital, Lanzhou, China; Universitá Sapienza di Roma, ITALY

## Abstract

**Objective:**

The application of robotic surgery for rectal cancer is increasing steadily. The purpose of this meta-analysis is to compare pathologic outcomes among patients with rectal cancer who underwent open rectal surgery (ORS) versus robotic rectal surgery (RRS).

**Methods:**

We systematically searched the literature of EMBASE, PubMed, the Cochrane Library of randomized controlled trials (RCTs) and nonrandomized controlled trials (nRCTs) comparing ORS with RRS.

**Results:**

Fourteen nRCTs, including 2711 patients met the predetermined inclusion criteria and were included in the meta-analysis. Circumferential resection margin (CRM) positivity (OR: 0.58, 95% CI, 0.29 to 1.16, *P* = 0.13), number of harvested lymph nodes (WMD: −0.31, 95% CI, −2.16 to 1.53, *P* = 0.74), complete total mesorectal excision (TME) rates (OR: 0.93, 95% CI, 0.48 to 1.78, *P* = 0.83) and the length of distal resection margins (DRM) (WMD: −0.01, 95% CI, −0.26 to 0.25, *P* = 0.96) did not differ significantly between the RRS and ORS groups.

**Conclusion:**

Based on the current evidence, robotic resection for rectal cancer provided equivalent pathological outcomes to ORS in terms of CRM positivity, number of harvested lymph nodes and complete TME rates and DRM.

## Introduction

Colorectal cancer accounts for approximately 10% of diagnosed cancers and cancer-related deaths worldwide each year [1]. The incidence of colorectal cancer worldwide is predicted to increase to 2.5 million in 2035 [1, 2]. Surgical resection plays a central role in the treatment of rectal cancer [3]. As one of the standard operations for low rectal cancer, abdominoperineal resection (APR), was introduced in the late nineteenth century [4]. APR operations have evolved over time; the present-day total mesorectal excision (TME) for rectal cancer has been made possible based on a thorough understanding of pathological aspects of diseases and their relation to surgical anatomy of the rectum. TME involves excision of the tumor and surrounding fascia which produces a characteristic specimen [5, 6]. The principles of TME treatment combine anatomy, embryological origin of the hindgut and the pathological spread of rectal cancer. TME became the gold standard for curative resection based on clinical evidence demonstrating better local control and survival [7]. Neoadjuvant therapy and adjuvant chemotherapy can be used as adjuvant agents to improve the prognosis after surgery [8, 9]. Minimally invasive surgeries result in comparable outcomes to open procedures with decreased perioperative blood loss and shorter recovery times [10, 11]. Robotic surgery has enabled meticulous and precise mesorectal dissection in a previously irradiated rectum down to the pelvic floor; however, open resection remains the gold standard for rectal cancer surgery [5, 7]. Circumferential resection margin (CRM) negativity and complete TME are associated with lower local and distal recurrence rates and better long-term survival [12, 13]. However, for rectal cancer, the narrow pelvic cavity can be a limitation to open TME. Robotics technology has been postulated to provide higher precision and visibility which can help to achieve better total mesenterectomy with potential for reduced perioperative complications. The aim of this meta-analysis was to compare pathologic outcomes between robotic rectal resection (RRS) and open rectal resection (ORS) for patients with rectal cancer.

## Methods

### Literature search

We systematically searched EMBASE, PubMed and the Cochrane Library for relevant articles (up to May 1, 2019). We used a search strategy with a combination of the following MeSH terms: Da Vinci/robotic/robot-assisted/open, rectal/colorectal/total mesorectal excision. Search algorithms for each database are summarized in S1 Table. We attempted to identify other studies by manually searching reference lists of the identified reports.

### Study inclusion and exclusion criteria

We evaluated prospective randomized controlled trials (RCTs) and non-randomized controlled trials (nRCTs) that compared RRS with ORS for rectal cancer; eligible studies with at least one of the following outcomes of interest: CRM positivity, harvested lymph nodes, complete TME and the length of distal resection margin (DRM), were included. Repeat publications of data from the same hospital, absent outcome parameters of interest, review articles, and case reports were excluded.

### Data extraction and quality assessment

Study selection, evaluation, and data extraction were checked independently by two investigators. Any disagreement was resolved through consultation with a third author. The outcomes of interest were complete TME, CRM positivity, number of harvested lymph nodes, and DRM. For each study, characteristics were extracted if available. If certain outcomes of the included studies were not reported, the corresponding authors were contacted via e-mail. The Jadad score, total score from 0 (poor) to 5 (excellent), was used to assess the quality of RCTs [14], otherwise, the Newcastle-Ottawa scale (NOS), total score from 0 (poor) to 9 (excellent), was used to assess the quality of nRCTs [15]. A Jadad scale score ≥3 points for RCTs and NOS score ≥6 points for nRCTs were considered high quality.

### Statistical analysis

Weighted mean differences (WMD) with a 95% confidence interval (CI) and odds ratios (OR) with 95% CI were used for the statistical analysis of continuous and dichotomous variables, respectively. Statistical heterogeneity was assessed using the Chi-square test with significance set at *P* < 0.10, and heterogeneity was quantified using the *I*^*2*^ statistic. A fixed-effect model was used to pool data when statistical heterogeneity was not present. Otherwise, the random-effects model was selected. Subgroup analysis was performed to identify potential heterogeneity [16, 17]. A subgroup analysis was performed based on the publication time, country, the total number of patients, body mass index (BMI) and neoadjuvant therapy used for all the measured outcomes. Begg’s and Egger’s tests were used to assess publication bias [18]. All analyses were performed using STATA/SE version 12.0 and Review Manager Version 5.3 (The Cochrane Collaboration, Oxford, London, UK). *P* < 0.05 was considered significant.

## Results

### Study characteristics

**Fig 1:**
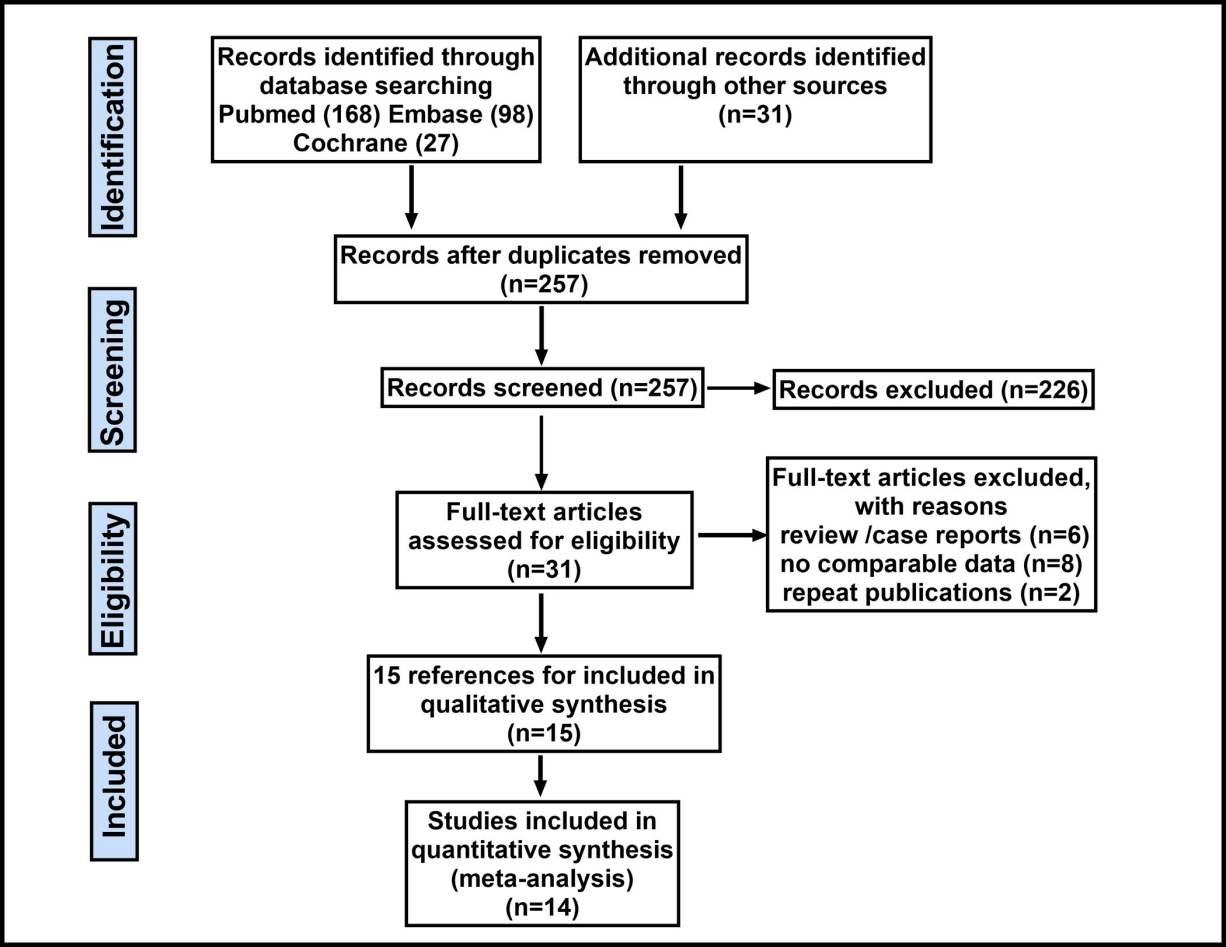
Flow diagram of the search results and study selection process.

Our initial search identified 324 citations of potentially eligible studies; 67 of these were excluded due to duplication and 226 were removed after screening the titles and abstracts. Overall, 31 potentially relevant articles were retrieved for full-text review (Fig 1) and of these, 14 nRCT studies [19–32], including 2711 patients (1123 patients who underwent RRS and 1588 patients who underwent ORS) fulfilled our eligibility criteria and were included in this systematic review. Examination of the references given in these studies did not provide any new eligible articles for assessment. The characteristics of patients included in the studies are summarized in Table 1. The characteristics of excluded prospective studies are summarized in S2 Table. The quality assessment of included studies using the Newcastle-Ottawa scale are summarized in S3 Table.

**Table 1:** Characteristics of the studies included in the meta-analysis.

Study	Country	RRS vs ORS													Follow-up (months)	Study quality*
		Number of patients	Male gender (%)	Age (year) (mean ± SD)	BMI (kg/m^2^) (mean ± SD)	Neoadjuvant Therapy (%)	Tumor to anal verge (cm), (mean ± SD)	Previous laparotomies (%)	Clinical tumor staging Ⅲ-Ⅳ (%)	learning curve cases Yes/No	pCR after neoadjuvant therapy (%)	Number of metastatic LN harvested	Lymphovascular invasion (%)	Procedure type (LAR:APR)		
Bertani, E. 2011	Italy	52 vs 34	60 vs 59	59.6 ± 11.6 vs 63.2 ± 10.5	24.8 ± 3.62 vs 25.6 ± 3.85	46 vs 44	8.4 (3–20) vs 9.7 (3–25)^†^	8.46 vs 35.3	40.3 vs 26.5	No	NA	0 (0–13) vs 0 (0–13)^†^	NA	NA	NA	5
deSouza, AL. 2011	USA	36 vs 46	61.1 vs 54.3	63.5 ± 11.5 vs 63.7 ± 12.1	27.4 (17.6–40.0) vs 28.7 (17.3–43.1)^†^	69.4 vs 50	NA	NA	27.8 vs 41.3	Yes	NA	NA	NA	29:7 vs 45:1	18	7
Biffi, R. 2011	Italy	49 vs 105	55.1 vs 60.0	59.5 ± 11.3 vs 61.6 ± 11.7	24.9 ± 4.0 vs 24.9 ± 3.6	73.5 vs 52.4	6.9 ± 2.6 vs 7.9 ± 3.4	NA	36.7 vs 40.0	Yes	NA	NA	NA	49:0 vs 105:0	NA	6
Park, JS. 2011	Korea	52 vs 88	53.8 vs 64.8	57.3 ± 12.3 vs 62.3 ± 10.4	23.7 ± 2.4 vs 23.3 ± 3.0	23.1 vs 10.2	7.6 ± 3.4 vs 8.9 ± 3.8	17.3 vs 14.8	42.3 vs 32.9	Yes	NA	1.1 ± 2.0 vs 1.3 ± 2.5	NA	52:0 vs 82:6	NA	6
Kang, JC. 2013	Korea	165 vs 165	63.0 vs 66.7	61.2 ± 11.4 vs 59.2 ± 11.0	23.1 ± 2.8 vs 23.0 ± 3.0	23.6 vs 32.7	12.0 ± 4.9 vs 11.4 ± 5.5	14.5 vs 12.7	32.7 vs 33.3	Yes	2.4 vs 7.2	1.0 ± 2.3 vs 1.3 ± 3.2	23.6 vs 21.2	164:0 vs 148:9	22.4 (1–48)^†^	7
Barnajian, DP. 2014	USA	20 vs 20	60 vs 60	62 (44–82) vs 61 (40–80)^†^	22 (18–31) vs 22 (18–31)^†^	50 vs 60	5 (1.5–10) vs 6 (2–10)^†^	60 vs 65	50.0 vs 45.0	Yes	NA	NA	NA	15:5vs 15:5	NA	6
Kim Jin C. 2014	Korea	108 vs 114	59.3 vs 68.4	57 ± 11 vs 61 ± 9	23.7 ± 2.7 vs 23.2 ± 3	83.3 vs 45.4	3.8 ± 1.2 vs 4 ± 0.9	8.3 vs 15.8	33.3 vs 23.7	No	NA	1.3 ± 3.1 vs 0.6 ± 1.4	23.1 vs 9.6	108:0 vs 114:0	21 ± 9	7
Ghezzi TL, 2014	Brazil, Italy	65 vs 109	63.1 vs 56.0	64 ± 12 vs 64 ± 13	24.7 ± 3.6 vs 25.4 ± 3.6	72.3 vs 61.5	6.2 ± 2.7 vs 6.1 ± 2.3	38.5 vs 40.4	35.4 vs 35.8	Yes	9.2 vs 9.2	NA	10.8 vs 10.1	55:9 vs 93:11	60	7
De Jesusa M. 2016	Brazil	59 vs 200	61 vs 50	60 (79–28) vs 57 (18–83)^†^	12 ± 26.7 vs 14 ± 31.1	NA	NA	71.1 vs 51.2	NA	Yes	NA	1 (0–18) vs 1 (0–28)^†^	NA	NA	NA	6
Ramji 2016	Canada	26 vs 26	73 vs 58	62.1 ± 9.1 vs 69 ± 12	27.8 ± 5.5 vs 27.9 ± 5.2	58 vs 23	8.6 ±7.9 vs 13.1 ± 7.5	NA	11.5 vs 34.6	No	NA	NA	NA	22:4 vs 20:6	NA	4
Yamaguchi, 2016	Japan	85 vs 88	77.6 vs 77.3	63 (36–78) vs 63 (26–84)^†^	22.8 (16.7–29.7) vs 23.3 (16.2–30.2)^†^	11.8 vs 12.5	5 (0–7) vs 5 (0–9)^†^	5.9 vs 14.8	77.6 vs 63.6	No	NA	NA	NA	72:13 vs 59:29	NA	6
Silva-Velazco, 2017	USA	66 vs 304	75.8 vs 69.4	59 (29–77) vs 58 (27–93)^†^	29.5 (22–66) vs 28 (15–57)^†^	51.5 vs 40.8	6.7 (0–15) vs 6 (0–15)^†^	19.7 vs 21.1	42.4 vs 53.9	Yes	12.1 vs 10.5	NA	NA	43:22 vs 187:98	NA	5
Ishihara, S. 2018	Japan	130 vs 234	58 vs 65	61.3 vs 64.1	22.2 vs 22.6	55.2 vs 62.1	NA	NA	NA	Yes	8.7 vs 4.7	NA	NA	130:0 vs 234:0	NA	6
Garfinkle, R. 2019	Canada	154 vs 211	68.8 vs 60.2	61.9±13.5vs 63.4 ±12.2	28.0 ±6.1 vs 28.7 ±6.4	NA	NA	NA	44.8 vs 44.1	No	NA	NA	NA	86:68 vs 99:112	NA	6

^**†**^ Reported as medians and range;

*Based on the Newcastle-Ottawa Scale (range 1–9 stars); ORS = open rectal surgery; RRS = robotic rectal surgery; BMI = body mass index; NA = not available. SD = standard deviation; LAR = Low anterior resection; APR = Abdominoperineal resection. pCR, pathological complete remission; Cancer staging according to the American Joint Committee on Cancer (7th ed., 2010).

### CRM positivity

**Fig 2:**
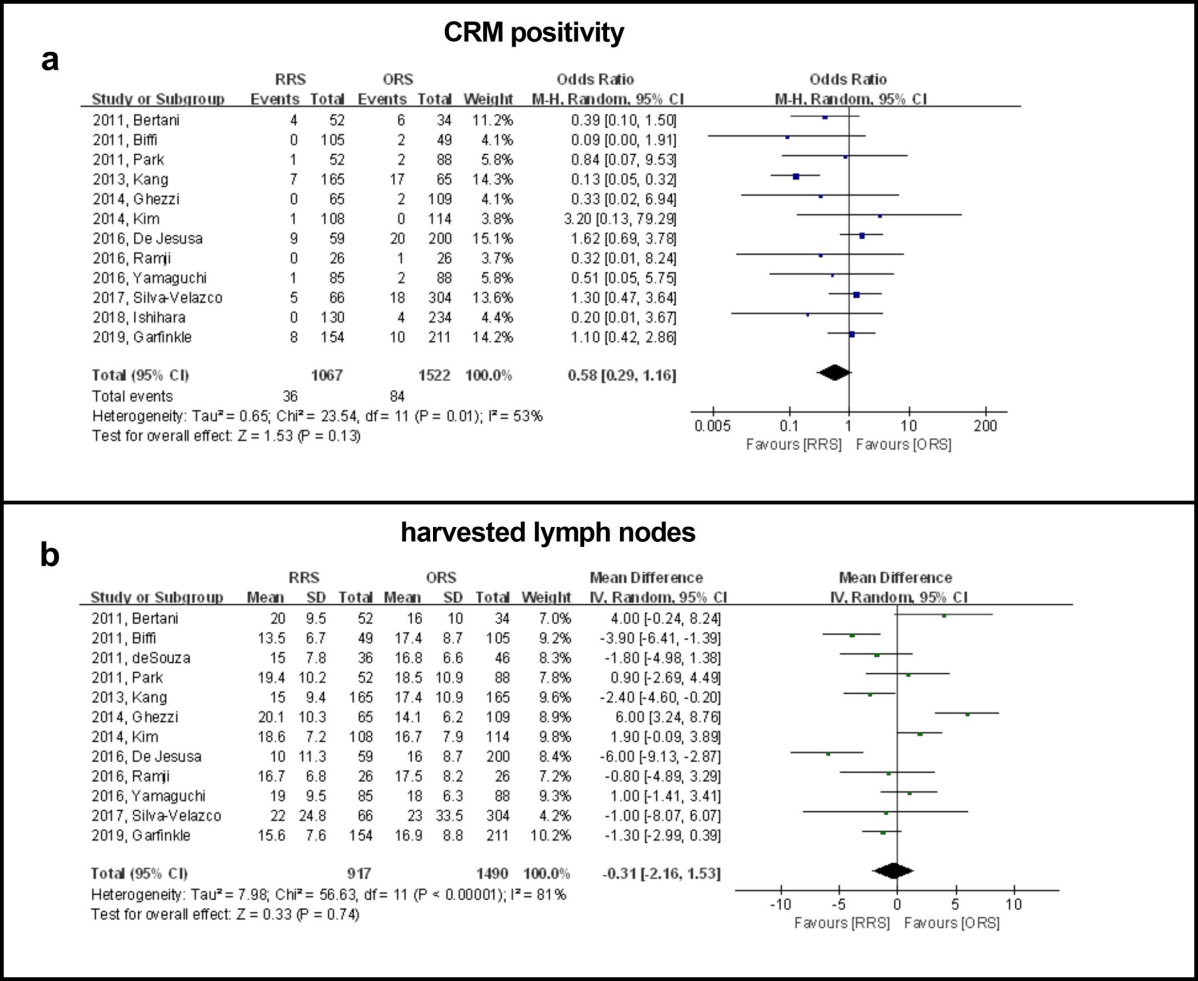
Forest plot showing RRS and ORS group comparisons in terms of CRM positivity and number of harvested lymph nodes.

Data on CRM positivity was available in 12 studies including 2589 patients and the results showed that there was no significant difference between the RRS and ORS groups (OR: 0.58, 95% CI, 0.29 to 1.16, *P* = 0.13) (Fig 2A). The subgroup analysis showed that publication time and country/geographic region might confound the prediction of CRM positivity based on differences in OR between the subgroups. The subgroup analyses performed according to the publication time revealed that the RRS group had a significantly higher CRM positivity rate when compared with the ORS group before 2014 (OR: −0.25, 95% CI: 0.13 to 0.48, *P* < 0.0001). An assessment based on country/geographic region revealed that the RRS group had a significantly lower CRM positivity rate when compared with the ORS group in Asian countries (OR: 0.24, 95% CI: 0.11 to 0.50, *P* = 0.0002) (Table 2).

**Table 2:** Subgroup analyses of pathologic outcomes based on the publication time, country, BMI, sample size and neoadjuvant therapy.

Outcome	Subgroup		No. of studies	No. of patients	Study Heterogeneity		Model	Meta-analysis	
				RRS vs ORS	*I*^*2*^ (%)	*P* Value*		WMD/OR (95% CI)	*P* Value*
Positive CRM	Year	Before 2014	6	547 vs 459	21	0.27	Fixed	-0.25^†^ (0.13, 0.48)	**< 0.0001**
		After 2014	6	520 vs 1063	0	0.67	Fixed	1.10^†^ (0.66, 1.82)	0.71
	Countries	Asia	5	540 vs 589	31	0.22	Fixed	0.24^†^ (0.11, 0.50)	**0.0002**
		Non-Asian	7	527 vs 933	13	0.33	Fixed	0.94^†^ (0.58, 1.52)	0.61
	Male (%)	≤ 60	8	621 vs 831	1	0.42	Fixed	0.90^†^ (0.54, 1.51)	0.70
		> 60	4	470 vs 668	79	0.003	Random	0.55^†^ (0.15, 1.98)	0.36
	BMI	≤ 25	9	821 vs 981	58	**0.01**	Random	0.45^†^ (0.17, 1.14)	0.09
		> 25	3	246 vs 541	0	0.72	Fixed	1.10^†^ (0.56, 2.19)	0.78
	Sample size	≤ 100	2	78 vs 60	0	0.91	Fixed	0.38^†^ (0.11, 1.31)	0.12
		> 100	10	989 vs 1462	60	0.007	Random	0.62^†^ (0.28, 1.39)	0.25
	Neoadjuvant	≤ 50%	3	300 vs 392	0	0.84	Fixed	0.18^†^ (0.03, 1.05)	0.06
		> 50%	9	767 vs 1130	62	0.007	Random	0.69^†^ (0.32, 1.48)	0.34
No. of lymph nodes	Year	Before 2014	7	527 vs 661	85	**< 0.00001**	Random	0.57 (-2.12, 3.26)	0.68
		After 2014	4	390 vs 829	67	**0.02**	Random	-1.62 (-4.03, 0.79)	0.19
	Countries	Asia	4	410 vs 455	66	**0.03**	Random	0.31 (-1.78, 2.41)	0.77
		Non-Asian	8	507 vs 1035	85	**< 0.00001**	Random	-0.66 (-3.44, 2.13)	0.64
	Male (%)	≤ 60	8	447 vs 722	86	**< 0.00001**	Random	0.01 (-2.84, 2.86)	1.00
		> 60	4	470 vs 768	30	0.23	Fixed	-1.07 (-2.22, 0.09)	0.07
	BMI	≤ 25	8	635 vs 903	87	**< 0.00001**	Random	-0.12 (-2.52, 2.77)	0.93
		> 25	4	282 vs 587	0	0.98	Fixed	-1.33 (-2.70, 0.05)	0.06
	Sample size	≤ 100	3	114 vs 106	58	0.09	Random	-0.28 (-3.14, 3.69)	0.87
		> 100	9	803 vs 1384	85	**< 0.00001**	Random	-0.50 (-2.70, 1.71)	0.66
	Neoadjuvant	≤ 50%	3	150 vs 260	93	**< 0.00001**	Random	0.10 (-6.03, 6.23)	0.98
		> 50%	9	767 vs 1230	71	**0.0005**	Random	-0.48 (-2.25, 1.30)	0.60
Complete TME	Year	Before 2014	1	20 vs 20	-	-	-	0.21^†^ (0.02, 2.08)	0.18
		After 2014	2	85 vs 323	0	0.84	Fixed	1.12^†^ (0.55, 2.27)	0.76
	Countries	Asia	0	-	-	-	-	-	-
		Non-Asian	3	105 vs 343	0	0.39	Fixed	0.93^†^ (0.48, 1.78)	0.83
	Male (%)	≤ 60	2	39 vs 39	27	0.24	Fixed	0.64^†^ (0.22, 1.88)	0.42
		> 60	1	66 vs 304	-	-	-	1.17^†^ (0.50, 2.75)	0.72
	BMI	≤ 25	1	20 vs 20	-	-	-	0.21^†^ (0.02, 2.08)	0.18
		> 25	2	85 vs 323	0	0.84	Fixed	1.12^†^ (0.55, 2.27)	0.76
	Sample size	≤ 100	2	39 vs 39	27	0.24	Fixed	0.64^†^ (0.22, 1.88)	0.42
		> 100	1	66 vs 304	-	-	-	1.17^†^ (0.50, 2.75)	0.72
	Neoadjuvant	≤ 50%	2	85 vs 323	0	0.84	Fixed	1.12^†^ (0.55, 2.27)	0.76
		> 50%	1	20 vs 20	-	-	-	0.21^†^ (0.02, 2.08)	0.18
DRM	Year	Before 2014	4	173 vs 247	46	0.14	Fixed	-0.60 (0.26, 0.94)	**0.0005**
		After 2014	2	111 vs 114	0	0.78	Fixed	-0.82 (-1.21, -0.43)	**< 0.0001**
	Countries	Asia	2	137 vs 176	92	**0.0005**	Random	-0.17 (-1.44, 1.10)	0.79
		Non-Asian	4	147 vs 185	72	**0.01**	Random	0.15 (-0.81, 1.12)	0.75
	Male (%)	≤ 60	5	199 vs 273	75	**0.003**	Random	-0.40 (-1.16, 0.36)	0.30
		> 60	1	85 vs 88	-	-	-	-0.80 (-1.21, -0.39)	**0.0001**
	BMI	≤ 25	5	258 vs 335	88	**< 0.00001**	Random	0.17 (-0.70, 1.03)	0.71
		> 25	1	26 vs 26	-	-	-	-1.00 (-2.35, 0.35)	0.15
	Sample sizer	≤ 100	3	98 vs 80	0	0.43	Fixed	-0.20 (-0.81, 0.40)	0.51
		> 100	3	186 vs 281	94	**< 0.00001**	Random	0.22 (-0.94, 1.39)	0.71
	Neoadjuvant	≤ 50%	4	215 vs 236	77	**0.005**	Random	-0.36 (-1.32, 0.59)	0.46
		> 50%	2	69 vs 125	81	**0.02**	Random	-0.53 (-0.45, 1.51)	0.29

^**†**^ odds ratio;

* Statistically significant results are shown in bold; RRS = robotic rectal surgery; ORS = open rectal surgery; CRM = circumferential resection margin; TME = total mesorectal excision; DRM = distal resection margin; BMI = body mass index; WMD/OR = weighted mean difference/odds ratio; df = degrees of freedom; CI = confidence interval.

### Harvested lymph nodes

Data on harvested lymph nodes were available in 12 studies including 2407 patients. The results showed that there were no significant differences between the RRS and ORS groups for number of nodes harvested (WMD: −0.31, 95% CI, −2.16 to 1.53, *P =* 0.74) (Fig 2B). A subgroup analysis showed that published year, country/geographic region, BMI, sample size and neoadjuvant therapy were not causes of heterogeneity in view of the dissimilarity in weighted mean difference between subgroups (Table 2).

### Complete TME

**Fig 3:**
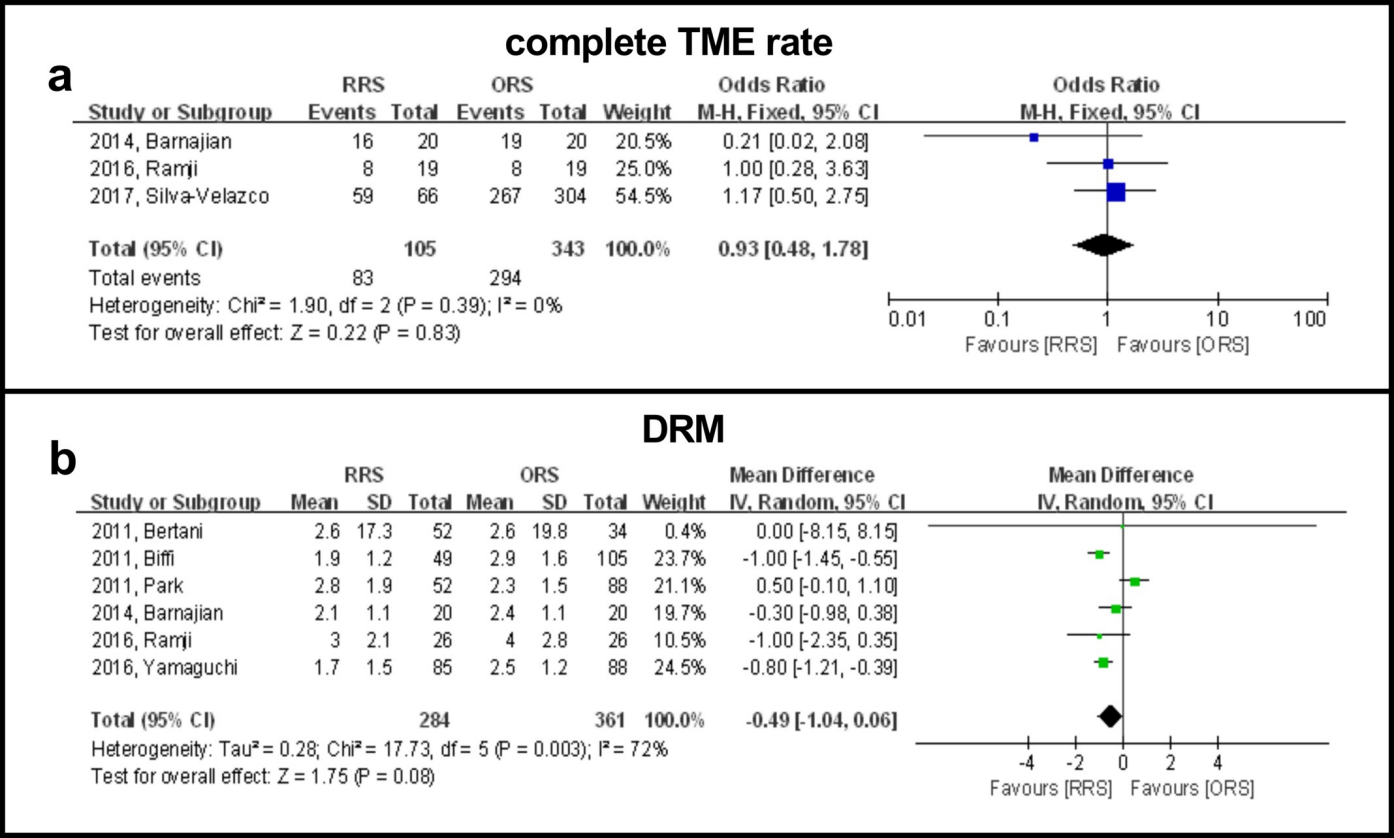
Forest plot showing RRS and ORS group comparisons in terms of complete TME rate and DRM.

Three studies including 377 patients reported complete TME and the results showed that there were no significant differences between the two groups (OR: 0.93, 95% CI, 0.48 to 1.78, *P* = 0.83) (Fig 3A). A subgroup analysis showed that published year, country/geographic region, BMI, sample size and neoadjuvant therapy were not causes of heterogeneity in view of the dissimilarity in weighted mean difference between subgroups (Table 2).

### DRM

Six studies reported the length of DRM in 645 patients and the results showed that there were no significant differences between the two groups (WMD: −0.49, 95% CI, −1.04 to 0.06, *P* = 0.96) (Fig 3B). A subgroup analysis showed that publication time was a possible cause of heterogeneity in view of the difference in WMD between subgroups. A subgroup analysis revealed that the RRS group had a significantly longer DRM than the ORS group when studies were completed before 2014 (WMD: 0.54, 95% CI: 0.03 to 1.06, *P* = 0.04). However, the RRS group had a significantly shorter DRM than the ORS group when studies were completed after 2014 (WMD: −0.82, 95% CI: −1.21 to −0.43, *P* < 0.0001) (Table 2).

### Sensitivity analysis and publication bias

**Fig 4:**
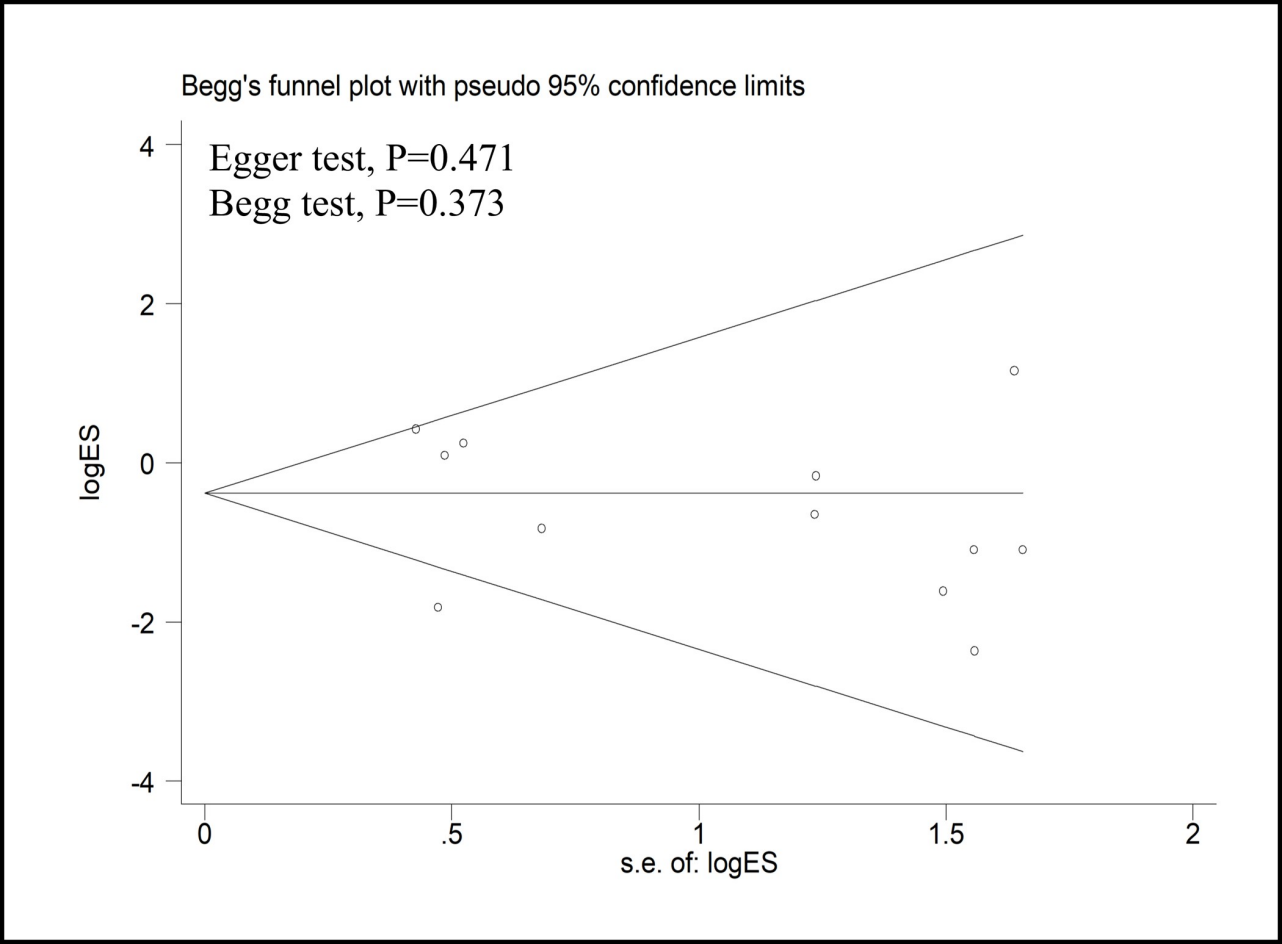
Funnel plots of the studies reporting on the CRM positivity rate. MD = mean difference; SE = standard error.

With the exception of three reports that scored five or fewer stars on the NOS, all other studies were included in the sensitivity analysis (Table 3). The sensitivity analysis was performed for CRM positivity, number of harvested lymph nodes, complete TME rate, and DRM. The results showed that there was no change in the significance of any of the outcomes. All of the above results suggested that the pooled outcomes were reliable. A funnel plot of the studies reporting on CRM positivity showed that there was no publication bias in the included studies (Egger’s test, *P* = 0.471; Begg’s test, *P* = 0.373) (Fig 4).

**Table 3:** Results of the sensitivity analysis of the pathologic outcomes.

Outcomes of interest	No. of studies	No. of patients	WMD/OR, (95% CI)	*P *value*	Study heterogeneity	
		RRS vs ORS			*I*^2^, %	*P *value*
Positive CRM	9	923 vs 1158	0.54^†^ (0.21, 1.36)	0.19	62	**0.007**
Harvested lymph nodes	9	773 vs 1126	-0.61 (-2.71, 1.50)	0.57	85	**<0.00001**
Complete TME	1	20 vs 20	0.21^†^ (0.02, 2.08)	0.18	-	-
DRM	4	206 vs 301	0.03 (-0.23, 0.29)	0.82	91	**<0.00001**

^**†**^ odds ratio;

* Statistically significant results are shown in bold; RRS = robotic rectal surgery; ORS = open rectal surgery; CRM = circumferential resection margin; TME = total mesorectal excision; DRM = distal resection margin; WMD/OR = weighted mean difference/odds ratio; df = degrees of freedom; CI = confidence interval.

## Discussion

Robotics is an emerging technology with wide acceptance in colorectal surgery [33]. This meta-analysis concluded that robotic resection for rectal cancer provided equivalent pathologic outcomes to the open approach in terms of CRM positivity, number of harvested lymph nodes, complete TME rate and DRM.

CRM positivity is minimized if the principle of TME is followed. Positive CRM positivity can be used as an alternative predictor of local recurrence, while intraoperative accidental perforation can be used as a predictor of adverse oncologic outcomes [34, 35]. One recently published review/meta-analysis showed that in RRS versus ORS groups, the CRM positivity rate was 3.4% and 5.5%, respectively and there were no significant differences [36]. Although not significant, the lower CRM positivity rate in the RRS group indicated a trend in favor of robotic rectal surgery, due to the well-illuminated magnified imaging of the pelvis provided with robotic surgery. A narrow pelvic space may have affected the pathological security of obese patients who underwent robotic rectal surgery because the advantages of robotic surgery could compensate for the difficulty. However, we did not find a higher CRM positivity rate in overweight patients with rectal cancer in our meta-analysis. These results suggest that being overweight does not directly influence the pathological outcome in patients with rectal cancer who undergo robotic surgery. It is interesting to note that the CRM positivity of the RRS group was lower than that of the ORS group in Asian countries while similar results were not observed in the European and American countries (Table 2).

A recent meta-analysis has identified significantly longer distal margins with the robotic approach compared with the open approach [36]. The present meta-analyses included more studies and did not demonstrate a significantly lower incidence of involved distal resection margins in the robotic approach compared with the open approach, however, the subgroup analysis showed a potential robotic advantage for male patients in term of DRM. The number of lymph nodes harvested is considered another prognostic indicator. The present meta-analysis, there was no significant difference in the number of lymph nodes obtained between the two groups, which was consistent with the results of one recently published meta-analysis [36]. The integrity of mesorectal resection is also an important indicator to evaluate the safety of rectal surgery for tumor resection and to predict recurrence in the pelvis [37]. As shown in one previous meta-analysis, the rate of complete TME was found to be similar between the RRS and ORS groups. These findings based on three studies focused on the pathologic outcomes of complete TME.

The length of the DRM is also thought to reflect the surgical quality and affect long-term oncological outcomes. We evaluated the quality of mesorectal excision with DRM parameters and found no significant difference between the two procedures. These results are considered oncologically acceptable [38, 39]. Surprisingly, the mean DRM length of the RRS group was shorter than that of the ORS group based on studies completed after 2014. The rate of abdominoperineal resection from one study [31] of this meta-analysis was higher with the open approach; therefore the length of the distal margin was longer in the ORS group, and this study indicated that the length of the DRM of the sphincter-preserving procedure was 17 mm and that of the abdominoperineal resection was 43 mm [31]. The surgeons mentioned in nine studies included in this meta-analysis were also affected by the learning curve for robotic rectal surgery. Robotic rectal surgery is technically challenging and has a steeper learning curve because it requires not only precise tumor margin resection but complex and time-dependent intestinal reconstruction. To overcome the learning curve of robotic colorectal surgery, experience with between 15 and 25 surgeries is required to develop proficiency [40]. Unfortunately, the included studies did not explain whether the surgeon has overcome the learning curve, and surgeons with less experience in robotic surgery may have a potential impact on outcomes.

The remote control, along with the placement of wristed instruments in line with pelvic walls, allows the surgeon to perform the rectal resection much more ergonomically [41, 42]. However, we did not find differences in terms of oncologic outcomes between the robotic and open approaches. The reason may be that the same oncologic principles for lymphovascular pedicle division and the extent of colorectal resection were applied [43]. Another meta-analysis, based on seven prospective and retrospective studies, that did not include the most recently published studies, showed no differences in terms of oncologic outcomes between the two approaches [44]. For example, there was no significant difference in pathological outcomes between robotic and laparoscopic rectal cancer surgery, which was confirmed by our previous studies [45].

The following limitations of our study need to be considered. First, four clinical trials of this meta-analysis had a sample size of less than 100, which were typically small studies. Second, the substantial heterogeneity of the included studies may reduce the power of the results. Third, two groups were comparable in age, sex, body mass index, and tumor stage. According to our quality assessment, part of observational studies included were prone to risk of selection bias, which have a potential impact on outcomes. However, subgroup analyses did not show significantly different results, supporting the findings of this meta-analysis. This study was conducted at an appropriate time, because enough data was available to use meta-analytical methods which enabled us to provide the most up-to-date information on this topic.

In conclusion, our study showed that the robotic resection for rectal cancer provided equivalent pathological outcomes to the open approach in terms of CRM positivity, number of harvested lymph nodes, complete TME rate and DRM. Future, large well-designed RCTs, are warranted to confirm the findings of this study.

## Supporting information

S1 Fig Forest plot of RRS and ORS group comparisons for anastomotic leakage. (PDF)

S2 Fig Forest plot of RRS and ORS group comparisons for ileus. (PDF)

S3 Fig Forest plot of RRS and ORS group comparisons for abdominal abscess formation. (PDF)

S4 Fig Forest plot of RRS and ORS group comparisons for wound infections. (PDF)

S5 Fig Forest plot of RRS and ORS group comparisons for pneumonia. (PDF)

S6 Fig Forest plot of RRS and ORS group comparisons for urinary infections. (PDF)

S7 Fig Forest plot of RRS and ORS group comparisons for overall morbidity. (PDF)

S8 Fig Forest plot of RRS and ORS group comparisons for mortality. (PDF)

S1 Table Search algorithms for each database. (DOCX)

S2 Table Characteristics of excluded prospective studies. (DOCX)

S3 Table The quality assessment of included studies using the Newcastle-Ottawa scale. (DOCX)

S1 Checklist PRISMA 2009 checklist. (DOC)
