# Supplementary figures and images for: Comparison of pathologic outcomes of robotic and open resections for rectal cancer: A systematic review and meta-analysis
Source: PLoS One. 2021 Jan 13;16(1):e0245154. doi: 10.1371/journal.pone.0245154 (PMC7806147; doi:10.1371/journal.pone.0245154)

# anastomotic leakage

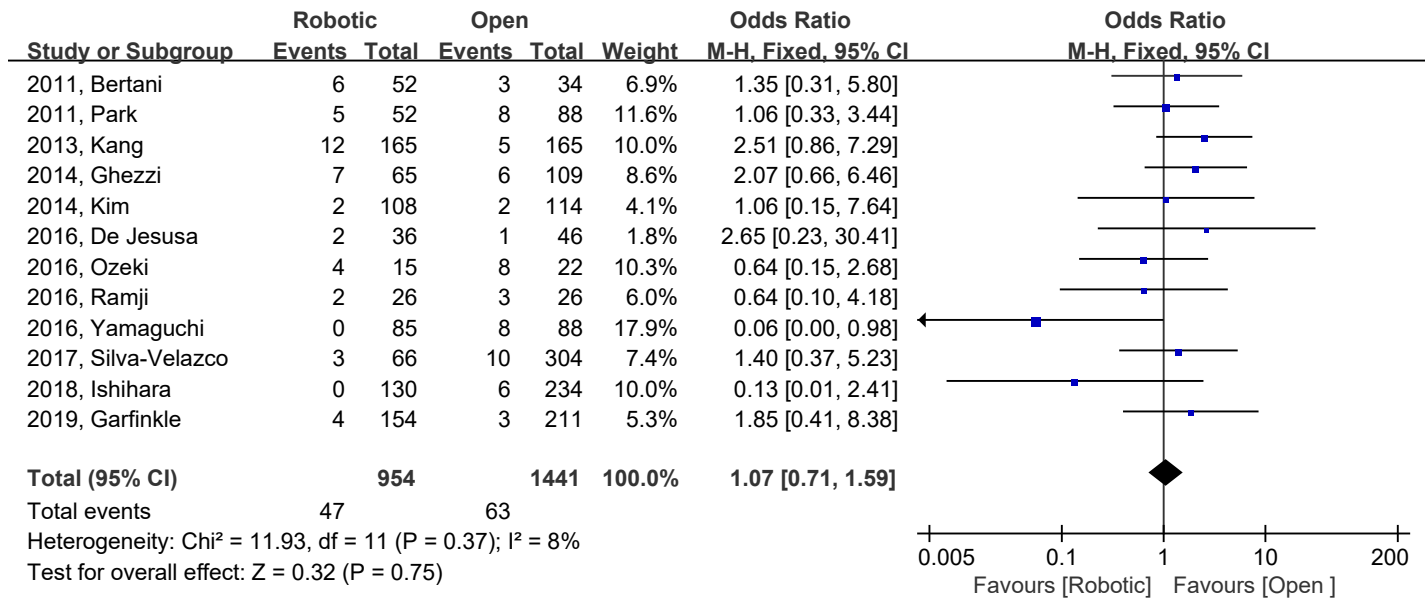

Supplement: S1 Fig — (PDF) [file pone.0245154.s001.pdf]

# ileus

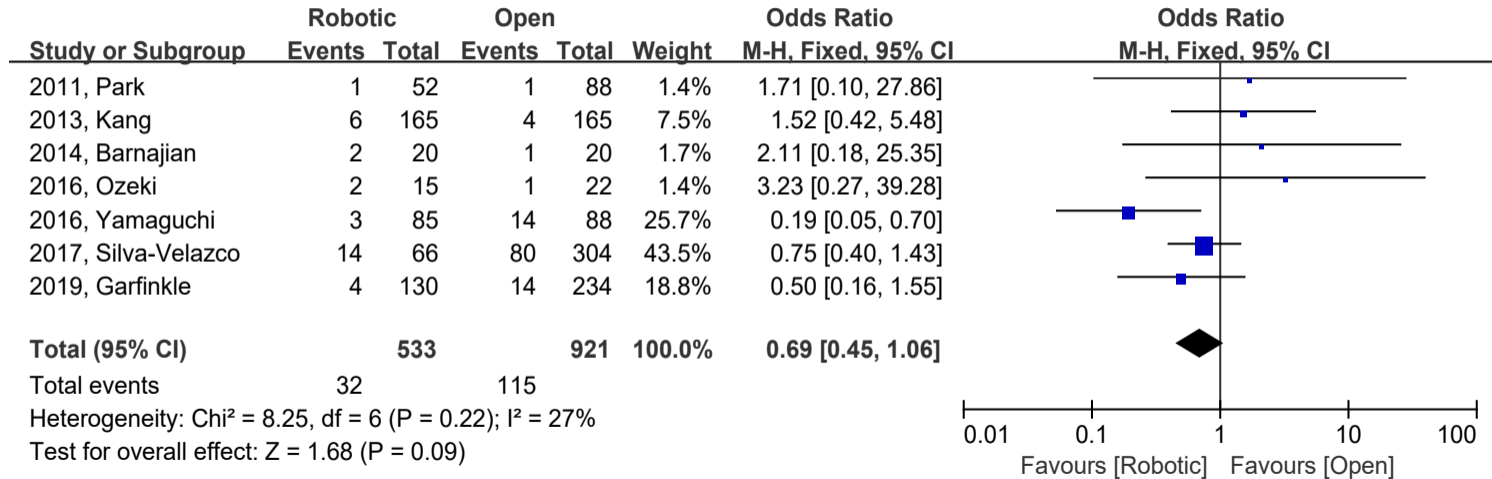

Supplement: S2 Fig — (PDF) [file pone.0245154.s002.pdf]

## abdominal abscess

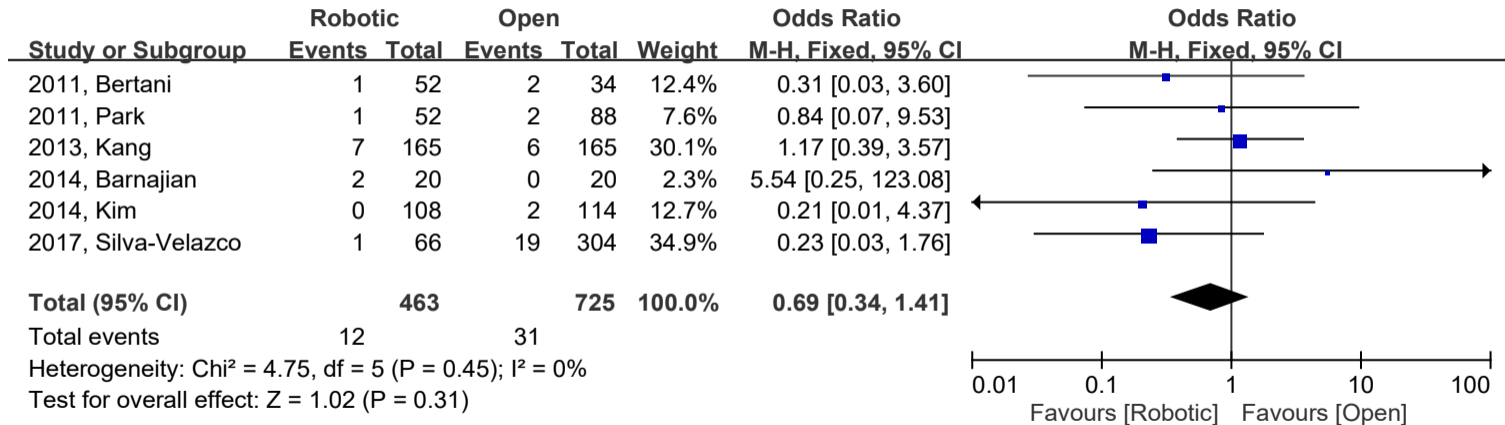

Supplement: S3 Fig — (PDF) [file pone.0245154.s003.pdf]

## wound infection

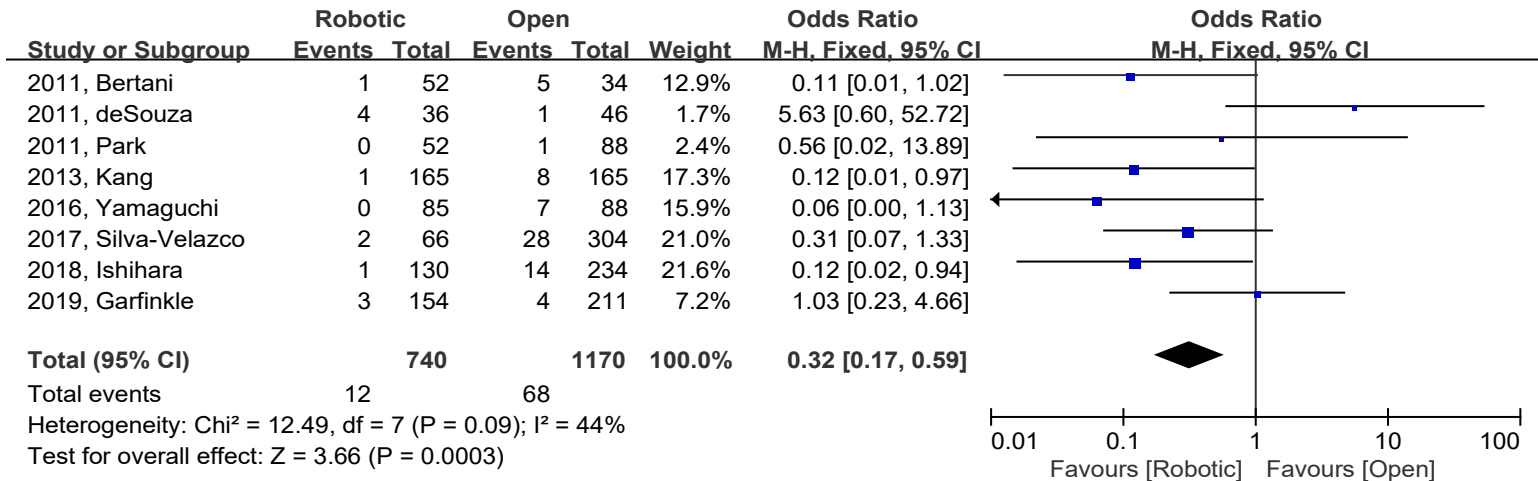

Supplement: S4 Fig — (PDF) [file pone.0245154.s004.pdf]

## urinary infection

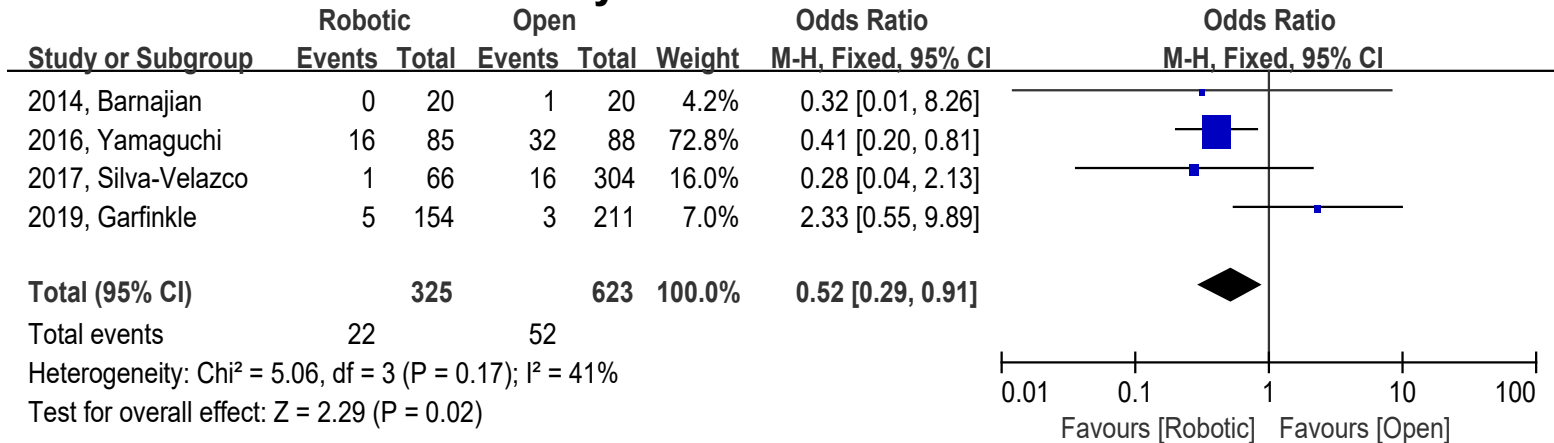

Supplement: S6 Fig — (PDF) [file pone.0245154.s006.pdf]

## overall morbidity

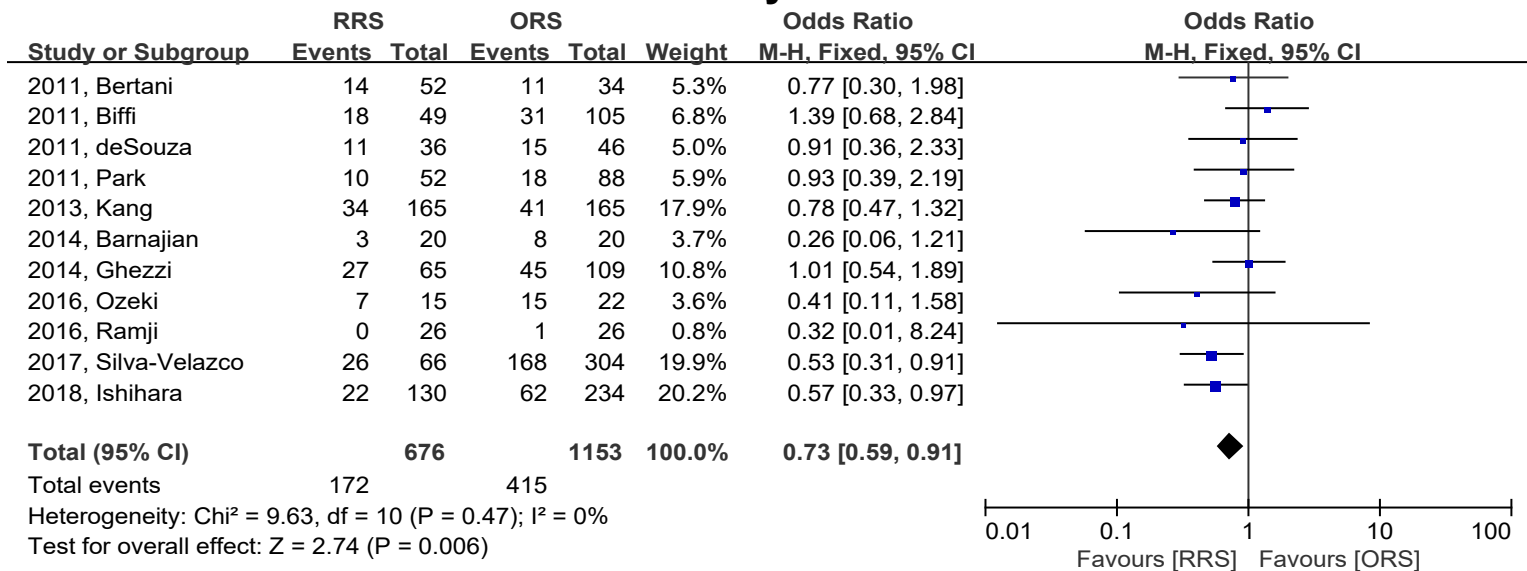

Supplement: S7 Fig — (PDF) [file pone.0245154.s007.pdf]

# mortality

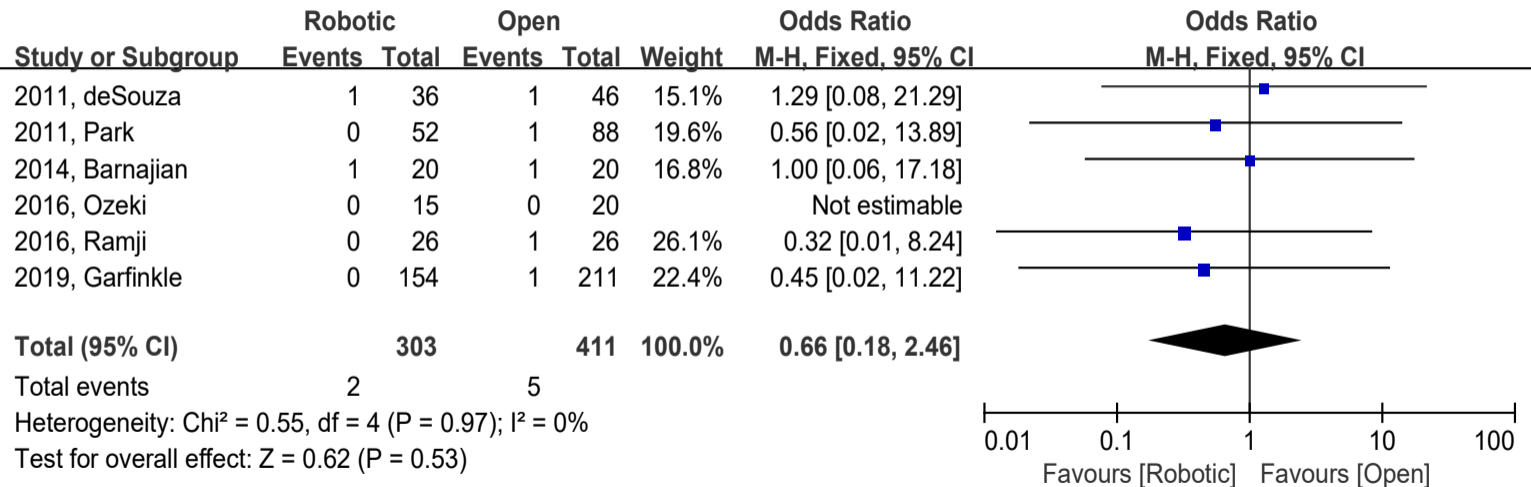

Supplement: S8 Fig — (PDF) [file pone.0245154.s008.pdf]
