# Supplementary material for: Comparison of pathologic outcomes of robotic and open resections for rectal cancer: A systematic review and meta-analysis
Source: PLoS One. 2021 Jan 13;16(1):e0245154. doi: 10.1371/journal.pone.0245154 (PMC7806147; doi:10.1371/journal.pone.0245154)

pneumonia

Study or Subgroup	Robotic Events	Robotic Total	Open Events	Open Total	Weight	Odds Ratio M-H, Fixed, 95% CI
2011, deSouza	2	36	0	46	23.0%	6.74 [0.31, 144.90]
2017, Silva-Velazco	0	66	1	304	30.0%	1.52 [0.06, 37.76]
2019, Garfinkle	1	154	1	211	47.0%	1.37 [0.09, 22.12]
**Total (95% CI)**		**256**		**561**	**100.0%**	**2.65 [0.53, 13.28]**
Total events	3		2			

Heterogeneity: Chi² = 0.69, df = 2 (P = 0.71); I² = 0%

Test for overall effect: Z = 1.19 (P = 0.24)

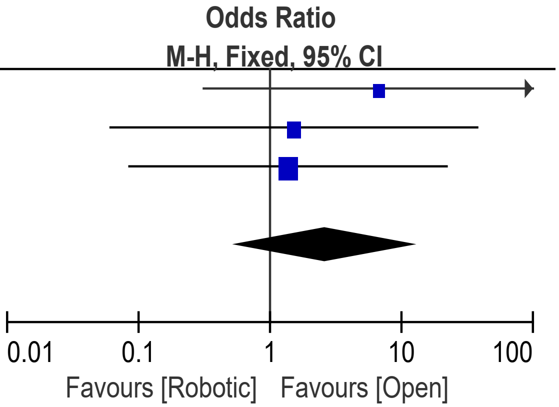

Supplement: S5 Fig — (PDF) [file pone.0245154.s005.pdf]
