# Supplementary material for: Comparison of pathologic outcomes of robotic and open resections for rectal cancer: A systematic review and meta-analysis
Source: PLoS One. 2021 Jan 13;16(1):e0245154. doi: 10.1371/journal.pone.0245154 (PMC7806147; doi:10.1371/journal.pone.0245154)
Supplement: S1 Table — (DOCX) [file pone.0245154.s009.docx]

**S1 Table.** **Search algorithms for each database**

| **Database** | **Search strategy** |
| --- | --- |
| **PubMed** | ((((conventional[Title/Abstract]) OR (conventionals[Title/Abstract])) OR (open[Title/Abstract])) AND (((Da Vinci[Title/Abstract]) OR (robotic[Title/Abstract])) OR (robot-assisted[Title/Abstract]))) AND ("Rectal Neoplasms" [Mesh]) OR ((((((((((((((Neoplasm*, Rectal [Title/Abstract]) OR Rectal Neoplasm* [Title/Abstract]) OR Rectal Tumor* [Title/Abstract]) OR Rectal Cancer* [Title/Abstract]) OR Rectum Cancer* [Title/Abstract]) OR Neoplasm, Rectum [Title/Abstract]) OR Rectum Neoplasm [Title/Abstract]) OR Tumor, Rectal [Title/Abstract]) OR Cancer of Rectum [Title/Abstract]) OR Cancer, Rectal [Title/Abstract]) OR Cancer, Rectum [Title/Abstract]) OR Cancer of the Rectum [Title/Abstract])) |
| **Embase** | #1 rectum tumor ’ /exp.  #2 mass, rectum ’ :ab,ti OR ‘ pararectal tumor ’ :ab,ti OR ‘ pararectal tumor ’ :ab,ti OR ‘ rectal mass ’ :ab,ti OR ‘ rectal neoplasm* ’ :ab,ti OR ‘ rectal tumor ’ :ab,ti OR ‘ rectal tumor ’ :ab,ti OR ‘ rectum mass ’ :ab,ti OR ‘ rectum neoplasm ’ :ab,ti OR ‘ rectum tumor ’ :ab,ti OR ‘ retrorectal tumor ’ :ab,ti OR ‘ retrorectal tumor ’ :ab,ti OR ‘ tumor recti ’ :ab,ti  #3: #1 OR #2  #4 conventional ’ :ab,ti OR ‘ conventionals ’ :ab,ti OR ‘ open ’ :ab,ti  #5 Da Vinci ’ :ab,ti OR ‘ robotic ’ :ab,ti OR ‘ robot-assisted ’ :ab,ti  #6: #3 AND #4 AND #5 |
| **The Cochrane Library** | #1 MeSH descriptor: [Rectal Neoplasms] explode all trees  #2 Neoplasm, Rectum  #3 Rectal Cancer*  #4 Rectum Cancer*  #5 Rectum Neoplasm*  #6 Rectal Tumor*  #7 Neoplasm*, Rectal  #8 Cancer, Rectal  #9 Cancer of Rectum  #10 Tumor, Rectal  #11 Rectal Neoplasm  #12: #1 or #2 or #3 or #4 or #5 or #6 or #7 or #8 or #9 or #10 or #11  #13 #conventional  #14 #conventionals  #15 #open  #16: #17 or #18 or #19  #20 Da Vinci  #21 robotic  #22 robot-assisted  #23: #20 or #21 or #22  #24: #12 AND #16 AND #23 |
