# Supplementary material for: Comparison of pathologic outcomes of robotic and open resections for rectal cancer: A systematic review and meta-analysis
Source: PLoS One. 2021 Jan 13;16(1):e0245154. doi: 10.1371/journal.pone.0245154 (PMC7806147; doi:10.1371/journal.pone.0245154)
Supplement: S2 Table — (DOCX) [file pone.0245154.s010.docx]

**S2 Table.** Characteristics of excluded prospective studies

| Study | Design | Total number of patients | Reasons for exclusion |
| --- | --- | --- | --- |
| Zawadzki, M, 2017 | Prospective nonrandomized study | 79 (38 RRS, 41 ORS ) | Unanalyzable data |
| Langer, D, 2017 | Prospective nonrandomized study | 106 (53 RRS, 53 ORS ) | High rate of selection bias |
| Zawadzki, M, 2017 | Prospective nonrandomized study | 61 (30 RRS, 31 ORS ) | Unanalyzable data |
| Jayne, D, 2017 | Randomized controlled trial | 237 (237 RRS ) | Review |
| RRS=robotic rectal surgery; ORS=open rectal surgery. | | | |
