# Supplementary material for: Comparison of pathologic outcomes of robotic and open resections for rectal cancer: A systematic review and meta-analysis
Source: PLoS One. 2021 Jan 13;16(1):e0245154. doi: 10.1371/journal.pone.0245154 (PMC7806147; doi:10.1371/journal.pone.0245154)
Supplement: S3 Table — (DOCX) [file pone.0245154.s011.docx]

**S3 Table: The quality assessment of included studies using the Newcastle-Ottawa scale**

| Author/Year | **Selection** | | | | **Comparability** | | **Exposure** | | | Total quality score |
| --- | --- | --- | --- | --- | --- | --- | --- | --- | --- | --- |
|  | Is the case definition adequate? | Representativeness of the cases | Selection of Controls | Definition of Controls | study controls for harvested lymph nodes | study controls for positive CRM | Ascertainment of exposure | Same method of ascertainment for cases and controls | Non-Response rate |  |
| Bertani, E. 2011 | 1 | 1 | 1 | 1 | 1 | 0 | 0 | 0 | 0 | **5** |
| deSouza, AL. 2011 | 1 | 1 | 1 | 0 | 1 | 0 | 1 | 1 | 1 | **7** |
| Biffi, R. 2011 | 1 | 1 | 1 | 1 | 0 | 1 | 1 | 0 | 0 | **6** |
| Park, JS. 2011 | 1 | 0 | 0 | 1 | 1 | 1 | 1 | 0 | 1 | **6** |
| Kang,JC. 2013 | 1 | 1 | 1 | 0 | 1 | 0 | 1 | 1 | 1 | **7** |
| Barnajian, DP. 2014 | 1 | 0 | 0 | 1 | 1 | 0 | 1 | 1 | 1 | **6** |
| Kim.Jin C. 2014 | 1 | 1 | 1 | 1 | 1 | 0 | 1 | 1 | 0 | **7** |
| Ghezzi.TL, 2014. | 1 | 0 | 1 | 1 | 1 | 0 | 1 | 1 | 1 | **7** |
| De Jesusa , M. 2016 | 1 | 1 | 1 | 0 | 1 | 0 | 1 | 0 | 1 | **6** |
| Ramji.2016 | 1 | 1 | 1 | 1 | 0 | 0 | 0 | 0 | 0 | **4** |
| Yamaguchi, 2016 | 1 | 0 | 1 | 1 | 0 | 1 | 1 | 0 | 1 | **6** |
| Silva-Velazco, 2017 | 0 | 1 | 1 | 1 | 1 | 0 | 0 | 1 | 0 | **5** |
| Ishihara, S. 2018 | 1 | 1 | 0 | 1 | 1 | 1 | 1 | 0 | 0 | **6** |
| Garfinkle, R. 2019 | 1 | 0 | 1 | 0 | 1 | 1 | 1 | 0 | 1 | **6** |
